# Regulation of Cancer Metastasis by PAK2

**DOI:** 10.3390/ijms252413443

**Published:** 2024-12-15

**Authors:** Megan Wu, Chandan Sarkar, Bin Guo

**Affiliations:** 1 The Kinkaid School, Houston, TX 77024, USA; wumegan07@gmail.com; 2 Department of Pharmacy, Bangabandhu Sheikh Mujibur Rahman Science and Technology University, Gopalgonj 8100, Bangladesh; csarkar1053@gmail.com; 3 Department of Pharmacological and Pharmaceutical Sciences, University of Houston, Houston, TX 77204, USA

**Keywords:** PAK2, rho family GTPases, cell cycle, apoptosis, cancer metastasis

## Abstract

PAK2 is a serine-threonine kinase and a member of the p21-activated kinase (PAK) family. PAK2 is activated by GTP-bound rho family GTPases, Rac, and Cdc42, and it regulates actin dynamics, cell adhesion to the extracellular matrix, and cell motility. In various types of cancers, PAK2 has been implicated in the regulation of cancer cell proliferation, cell cycle, and apoptosis. In addition, recent studies have shown that PAK2 plays an important role in cancer cell metastasis, indicating PAK2 as a potential therapeutic target. This review discusses recent discoveries on the functions of PAK2 in the regulation of various types of cancers. A better understanding of the mechanisms of function of PAK2 will facilitate future development of cancer therapies.

## 1. Introduction

PAK2, a serine/threonine protein kinase, is a member of the p21-activated kinase family (PAK), which includes six proteins that are broadly classified into two groups: Group I enzymes of PAK1, PAK2, and PAK3 that are activated by GTP-bound rho family GTPases Ras-related C3 botulinum toxin substrate (Rac) and cell division control protein 42 (Cdc42), and Group II enzymes PAK4, PAK5, and PAK6 that have similar kinase domains to that of group I PAKs but are not activated by a rho GTPase [1]. The PAK enzymes have important functions in normal physiology and play a key role in many pathological conditions. For example, PAKs are known to regulate cytoskeletal remodeling, focal adhesion assembly, cell migration, cell cycle, cell death, as well as gene expression.

Among the two groups of PAKs, Group I PAKs are extensively studied, and their mechanisms of action are better understood. These PAK enzymes regulate gene transcription, protein synthesis, and cellular signaling in different tissues through the phosphorylation of protein substrates that control cytoskeletal remodeling, cell division, and cell death. Specifically for PAK2, its phosphorylation substrates include important proteins such as myosin light-chain kinase (MLCK, which regulates cytoskeletal dynamics) [2], myelocytomatosis oncogene (Myc, which regulates cell growth 3) [3], and mitogen-activated protein kinase–interacting kinase 1 (Mnk1, which regulates apoptosis) [4]. Additionally, even though Group I PAKs are broadly responsible for cell motility and morphology, PAK2 has some characteristics that are unique from its peers. Notably, PAK2 is ubiquitously expressed, unlike PAK1, which is primarily expressed in adulthood, or PAK3, which is largely expressed within the brain [5]. Each Group I PAK plays different roles in cell adhesion, whereas PAK1 regulates cell spreading, and PAK2 slows down cell attachment to surfaces. PAK2 regulates cell senescence and organismal aging by regulating gene expression and histone 3.3-nucleosome assembly [6]. PAK2 has been mainly studied for its role in apoptosis. PAK2 is cleaved by caspase-3 [7] and contributes to the induction of apoptosis. In contrast, full-length PAK2 inhibits apoptosis by phosphorylation of caspase-7 [8].

PAK2 expression is associated with advanced tumor progression and poor prognosis [9,10]. Currently, small molecule inhibitors are available to inhibit PAK2 with high specificity [11,12]. A better understanding of the functions of PAK2 in cancer progression will facilitate the development of PAK2-targeted therapeutics. This review focuses on the function of PAK2 in the regulation of the metastasis of various cancers.

## 2. Structure of PAK2 and Mechanisms of PAK2 Activation

**Figure 1:**
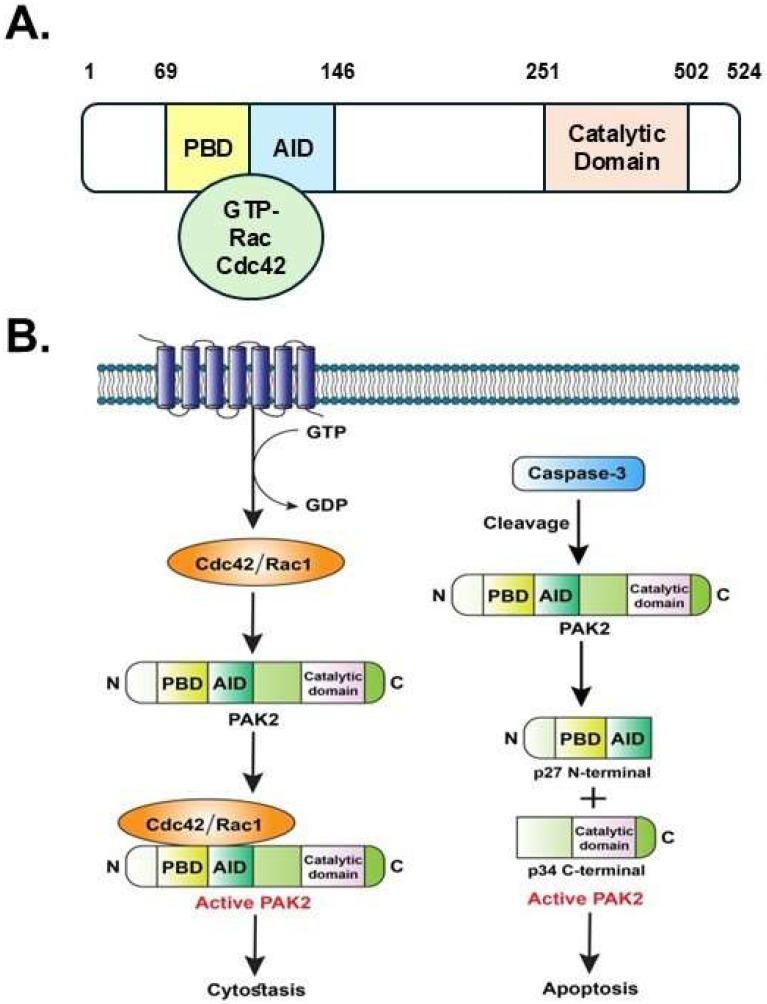
Structure and activation of PAK2. (**A**). The domain structure of the PAK2 protein. (**B**). Two different mechanisms that activate PAK2. AID: auto-inhibitory domain; Cdc42: cell division cycle 42, PBD: p21 binding domain; PAK2: p21-activated kinase 2; Rac1: Ras-related C3 botulinum toxin substrate 1.

Multiple PAKs, which are encoded by simple eukaryotes like yeasts and molds, function downstream of rho-family GTPases to control cytoskeletal structure and gene transcription, just as their orthologs in other systems [13]. The Group I PAKs share similar structures that have been evolutionarily conserved. The 524-amino-acid-long PAK2 protein with the molecular weight of 58 kDa has an N-terminal non-catalytic domain: the p21 binding domain (PBD), which binds to GTP-bound Rac and Cdc42 (Figure 1A). An auto-inhibitory domain (AID) partially overlaps with the PBD domain and forms the regulatory region together with PBD. The catalytic domain of the PAK2 kinase is in the C-terminal (amino acids 251–502). PAK2 exists as a homodimer and normally remains inactive due to the inhibitory interaction of the AID domain with the kinase domain of the dimer partner. Full-length PAK2 mainly presents in the cytoplasm, while the proteolytic PAK2-p34 fragment is found in the nucleus [14]. Notably, the distribution of PAK2 varies according to the stage of oocyte maturation: PAK2 is mostly found in the nucleus during the germinal vesicle (GV) stage. PAK2 is found in the cytoplasm and surrounding the chromosomes during GV breakdown. During stages I and II of metaphase, PAK2 is found in the cytoplasm and builds up in the primary microtubule organizing centers [15].

The binding of GTP-bound Cdc42 or Rac1 with the PBD of PAK2 changes the protein conformation to disrupt the inhibitory interaction, resulting in the activation of PAK2 (Figure 1B) [16,17]. Alternatively, PAK2 can be activated through a GTPase-independent mechanism (Figure 1B). For example, PAK2 can be activated by caspases during apoptosis [18]. Caspase-3 cleaves full-length PAK2 into two fragments: the p27 N-terminal regulatory domain and the p34 C-terminal catalytic domain [19,20]. Processing by caspase-3 enhances the autophosphorylation and further activation of PAK2 [21]. Interestingly, Cdc42-activated PAK2 mediates cytostasis, while caspase-3-activated PAK2 mediates apoptosis [22]. In fact, the binding of Cdc42 causes autophosphorylation in key kinase activation sites, which prevents the activation of PAK2 by caspase-3, enabling PAK2 to function as a molecular switch for cytostasis and apoptosis in response to different types and levels of stress.

## 3. PAK2 Signaling and Its Regulation of Cell Motility, Growth, and Survival

**Figure 2:**
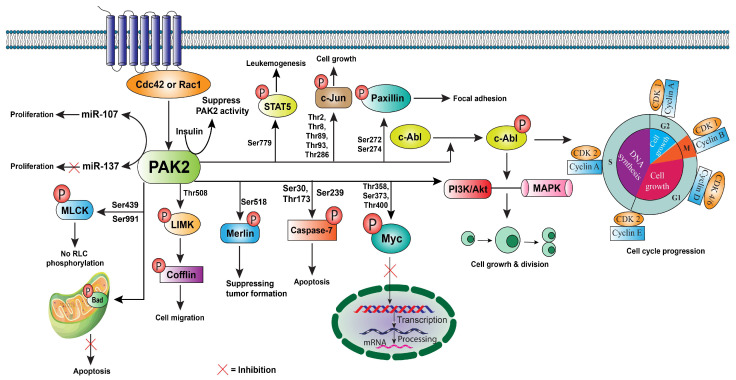
Phosphorylation of the substrate proteins by PAK2 regulates various cellular functions. PAK2 phosphorylates key proteins that are involved in cell cycle, apoptosis, and motility. Akt: Ak strain transforming; Bcl-2: B-cell lymphoma 2; Bad: Bcl-2 associated agonist of cell death; c-Abl: Abelson murine leukemia viral homolog 1; Cdc42: Cell division cycle 42, CDK2: cyclin-dependent kinase 2; LIMK: LIM kinase; MLCK: myosin light-chain kinase; MAPK: mitogen-activated protein kinase; miR: microRNA; Myc: myelocytomatosis oncogene; PI3K: phosphoinositide 3-kinases; Rac1: Ras-related C3 botulinum toxin substrate 1; RLC: regulatory light chains; STAT5: signal transducer and activator of transcription 5.

PAK2 can phosphorylate its substrate proteins to regulate their function in various cellular processes (Figure 2). The PAK2 substrates play important roles in cell proliferation, viability, and motility. For example, PAK2 can phosphorylate MLCK on serine residues 439 and 991 [2], which inhibits MLCK phosphorylation of myosin II regulatory light chains (RLC). MLCK plays a key role in cell migration by regulating cell membrane tension and protrusion [23]. Moreover, PAK2 phosphorylates LIM domain kinase (LIMK), which subsequently phosphorylates and deactivates cofilin, a protein that facilitates actin filament disassembly [24]. This process preserves actin stability at the leading edge, thereby enhancing cell migration. In addition, PAK2 also regulates focal adhesion formation by phosphorylating paxillin and influencing integrin signaling, which enhances cell attachment to the extracellular matrix (ECM) and supports stable migration [25].

PAK2 is also involved in regulating cell growth, primarily through its impact on signaling pathways that control the cell cycle and cell proliferation. One major pathway through which PAK2 influences cell growth is the mitogen-activated protein kinase (MAPK)/extracellular signal-regulated kinase (ERK) pathway [18]. PAK2 can activate the MAPK signaling cascade, which regulates various cellular responses, including growth and survival [26]. In parallel, activation of the phosphoinositide 3-kinase (PI3K)/protein kinase B (Akt) pathway provides pro-survival signals and promotes cell growth. Through PI3K activation, PAK2 indirectly promotes Akt phosphorylation, which supports growth and nutrient uptake by increasing mammalian target of rapamycin complex 1 (mTORC1) activity, essential for protein synthesis [27]. Moreover, PAK2 can phosphorylate protein tyrosine kinase Abelson murine leukemia viral homolog 1 (c-Abl) [28], a key regulator of cell cycle and cell growth. Phosphorylation by PAK2 stimulates c-Abl tyrosine kinase activity. In addition, PAK2 phosphorylates Myc protein, a key regulator of cell proliferation and growth, resulting in the inhibition of the ability of Myc to bind to DNA to activate transcription [3].

PAK2 plays a significant role in regulating cell survival by influencing several survival pathways that protect cells from apoptosis. One key mechanism by which PAK2 promotes cell survival is through its interaction with the anti-apoptotic protein Bcl-2. PAK2 activation of the PI3K/Akt pathway confers protection against apoptosis by phosphorylating the pro-apoptotic B-cell lymphoma 2 (Bcl-2) family protein Bad and inhibiting Bad-mediated apoptosis [29]. This action provides a survival advantage, particularly in cells undergoing oncogenic stress. Interestingly, in response to cellular stress such as DNA damage, PAK2 undergoes caspase-mediated cleavage, producing a C-terminal fragment that translocates to the nucleus [30]. This fragment has been shown to enhance apoptosis, suggesting that PAK2 can also act as a pro-apoptotic factor under certain conditions.

Transforming growth factor-β (TGF-β) is a potent activator of PAK2 [31]. This signaling molecule is involved in various cellular processes, including cell differentiation, migration, and apoptosis. The activation of PAK2 by TGF-β suggests a role for PAK2 in mediating these TGF-β-induced processes [13]. Moreover, *α*2-macroglobulin, when binding to glucose-regulated protein 78 (GRP78, a molecular chaperone), activates PAK2 [24]. This suggests that PAK2 may play a role in the cellular response to protein misfolding or stress. Furthermore, adenosine monophosphate-activated protein kinase (AMPK), a key energy sensor in cells, also activates PAK2 [32]. AMPK responds to changes in cellular energy status and regulates metabolic pathways, which might influence PAK2 in processes related to energy homeostasis and stress response [33]. The microRNAs, miR-107 and miR-137, are also known to regulate the expression of PAK2 [34,35]. miR-107 promotes cell proliferation through the PAK2 pathway [34], whereas miR-137 suppresses melanoma cell proliferation by targeting PAK2 [35].

On the other hand, PAK2 phosphorylates the tumor suppressor protein merlin at Ser518, which influences cell growth [36]. PAK2 also phosphorylates c-Jun at multiple sites, including Thr2, Thr8, Thr89, Thr93, and Thr286 [37]. These phosphorylations affect the activity of c-Jun, a transcription factor that regulates cell proliferation, survival, and stress response, thus influencing growth regulatory pathways. Moreover, PAK2 modulates apoptosis by phosphorylating caspase-7 at specific residues (Ser30, Thr173, and Ser239) [8]. Caspase-7 is involved in the execution phase of apoptosis, and PAK2′s influence on its activity suggests a role in regulating programmed cell death. PAK2 also phosphorylates paxillin at Ser272 and Ser274 [31]. Paxillin is a focal adhesion protein that plays a crucial role in cell migration and adhesion. Its phosphorylation by PAK2 may regulate the activation of proteases involved in extracellular matrix remodeling and cell movement [38]. Furthermore, PAK2 regulates signal transducer and activator of transcription 5 (STAT5) phosphorylation at Ser779, influencing leukemogenesis [39]. Additionally, phosphorylation of c-Myc at Thr358, Ser373, and Thr400 by PAK2 negatively regulates c-Myc’s ability to activate its target genes [3]. This inhibition suggests that PAK2 may act as a modulator of c-Myc activity, ensuring that its gene-stimulating effects are properly controlled [40].

Cellular stressors like hyperosmotic shock, UV light, and ionizing radiation activate caspase-dependent cleavage of PAK2, promoting apoptosis in various cell types [31]. In addition, insulin signaling has been shown to suppress PAK2 activity, enhancing glucose transporter protein type 4 (GLUT4)-mediated glucose uptake in neuronal cells [41].

## 4. Overexpression of PAK2 in Various Cancers

PAK2 overexpression has been extensively studied across different cancer types, revealing its multifaceted roles in tumor progression, metastasis, and resistance to therapies. Table 1 summarizes key findings from the literature, followed by critical analysis.

While PAK2 is widely implicated in tumorigenesis across diverse cancer types, its precise molecular mechanisms and downstream effectors vary significantly depending on the tumor context.

Across multiple cancers, PAK2 promotes cell proliferation, migration, invasion, and chemoresistance. These effects are often mediated through key signaling pathways like TGF-β, Akt, and β-catenin and transcriptional regulation via c-Myc [40,41,42,43,44,48]. In breast cancer, PAK2 modulates apoptosis via caspase-7 phosphorylation [8]. PAK2′s role in chemoresistance (e.g., HNC and ovarian cancer) suggests it as a potential therapeutic target. In prostate cancer, PAK2 enhances tumor plasticity by regulating SRY (sex-determining region Y)-box 2 (SOX2) expression, contributing to castration-resistant progression. However, its involvement in critical cellular pathways warrants careful targeting to avoid systemic toxicity [41]. In pancreatic and colorectal cancers, PAK2 interacts closely with the tumor microenvironment and cellular modulators like Mps one binder 1 (MOB1) and minichromosome maintenance complex component 7 (MCM7), emphasizing the need for context-specific therapeutic strategies [23,46,47,48]. Elevated PAK2 expression frequently correlates with poor survival outcomes, making it a promising prognostic marker. Its potential as a biomarker is reinforced by its correlation with aggressive phenotypes in gastric, renal, and ovarian cancers [10,42,43,44,49,52].

## 5. PAK2 Regulation of Cancer Metastasis

**Figure 3:**
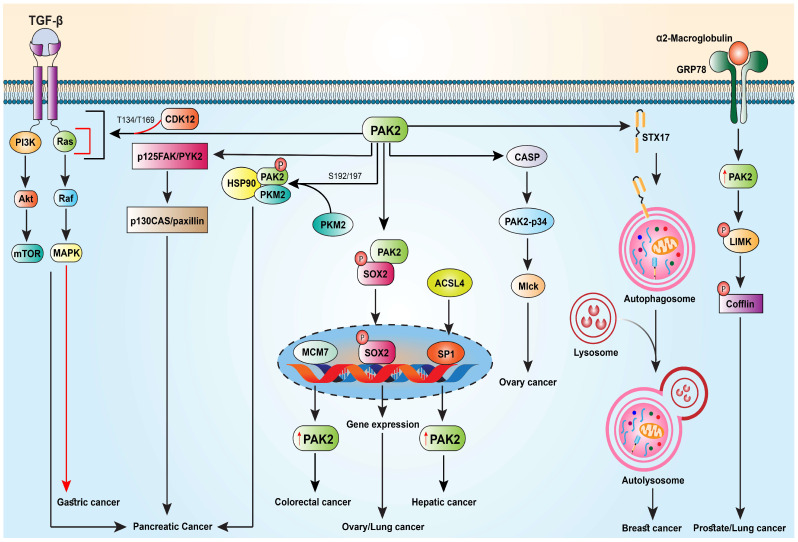
Mechanisms of action of PAK2 in the regulation of different signaling pathways in different cancers. The red arrow indicates that CDK12 activates the MAPK signaling pathway by directly binding to and phosphorylating PAK2 at T134/T169, thereby promoting gastric cancer progression. Upward arrows indicate the activation of PAK2. Akt: Ak strain transforming; ACSL4: Acyl-CoA synthetase long-chain family member 4; CDK2: cyclin-dependent kinase 12; CASP: caspase; FAK: focal adhesion kinase; PYK2: FAK-related proline-rich tyrosine kinase 2; HSP90: heat-shock protein 90; LIMK: LIM domain kinase; mTOR: mechanistic target of rapamycin; MAPK: mitogen-activated protein kinase; MCM7: minichromosome maintenance complex component 7; Mlck: myosin light-chain kinases; PI3K: phosphoinositide 3-kinases; PKM2: pyruvate kinase M2; Raf: rapidly accelerated fibrosarcoma; SOX2: SRY (sex-determining region Y)-box 2; STX17: Syntaxin 17.

Due to its function in regulating cancer cell survival, adhesion, and motility, PAK2 can play an important role in cancer metastasis. Recent studies have identified mechanisms of PAK2 in promoting metastasis in various cancers (Figure 3). These findings are summarized and discussed in different types of cancer (Table 2).

### 5.1. Breast Cancer

In clinical observations, PAK2 expression is associated with poor outcomes in patients with estrogen receptor-positive (ER+) breast cancer resistant to endocrine therapy, as it drives estrogen-independent tumor growth. Similarly, elevated PAK2 levels correlate with worse clinical outcomes in triple-negative breast cancer (TNBC) patients [54]. In preclinical studies, Lyu et al. demonstrated in TNBC cell lines that PAK2 inhibition using FRAX486 suppressed autophagy by degrading STX17. This impairment reduced TNBC cell migration and proliferation, suggesting a mechanistic role for PAK2 in metastasis [12].

### 5.2. Pancreatic Cancer

Like TNBC, pancreatic cancer is an incredibly lethal disease, and it has a 5-year survival rate of 6–8% [49]. Pancreatic cancer is also notoriously difficult to diagnose and detect despite the recent developments in the scientific understanding of its mechanisms. Elevated PAK2 levels are common in pancreatic cancer, where elevated levels of the protein activate the TGF-β signaling, which promotes the epithelial–mesenchymal transition process. Yang et al. sourced sequencing information from the Gene Expression Omnibus (GEO) to identify genes involved in pancreatic cancer metastasis and used a Gene Set Variation Analysis (GSVA) to determine the effects of pathway changes caused by these genes [49]. By conducting GSVA analysis on low- and high-expression PAK2 groups across cancer sites, Yang et al. found that the TGF-β signaling pathway is always upregulated. While causation seems to be tricky to establish, Yang et al.’s correlation analysis also demonstrated that PAK2 holds some significant relationship with the genes involved in the TGF-β signaling pathway. Regardless, this set of results seems to suggest that the TGF-β signaling pathway is the key route through which PAK2 works to cause pancreatic cancer. As a note, increased PAK2 levels have also been found to be correlated with other malignant traits such as angiogenesis and epithelial–mesenchymal transition. Finally, the statistical analysis also suggests that PAK2 reduces differentiation among pancreatic cancer cells, which increases their malignancy.

Furthermore, Cheng et al. demonstrated that pyruvate kinase M2 (PKM2) phosphorylates specific serine residues—Ser20, Ser141, and Ser192/197—on PAK2 in vitro, indicating that PKM2 serves as a novel regulator of PAK2 [55]. Their findings further revealed that PKM2-mediated phosphorylation of Ser192/197 plays an essential role in maintaining PAK2 levels within pancreatic ductal adenocarcinoma (PDAC) cells. Phosphorylation at Ser192/197 promotes PAK2 interaction with heat-shock protein 90 (*HSP90*), thereby preventing its ubiquitination and subsequent degradation by the proteasome. Additionally, their data suggested that phosphorylation at Ser141 and/or Ser20 may enhance the PKM2–PAK2 interaction. Collectively, these findings establish a novel regulatory pathway through which PKM2 modulates PAK2 activation by controlling its stability. Consequently, their study indicated that PKM2–PAK2 regulatory interactions are pivotal in driving metastasis in PDAC, underscoring the therapeutic potential of targeting the PKM2/HSP90/PAK2 complex in combating this aggressive malignancy.

In addition, research in rat pancreatic acini indicated that PAK2 plays a pivotal role in orchestrating the cellular signaling pathways essential for mediating various physiological and pathophysiological effects of cholecystokinin (CCK) [18]. The findings in this study demonstrate that, under physiological conditions, CCK-induced activation of PAK2 is critical for activating focal adhesion kinases (p125FAK and PYK2); adaptor and scaffold proteins (p130CAS and paxillin); MAPKs (ERK, JNK, and p42/44); and the PI3K/Akt signaling pathways—each known to mediate the effects of CCK in pancreatic acinar cells. Notably, they also showed that PAK2 is involved in pathophysiological events such as cell death and trypsin activation, which are implicated in the onset of pancreatitis. Together with evidence that inhibiting Rac1, an upstream activator of PAK, reduces the severity of pancreatitis, these results suggest that PAK2 may be a promising therapeutic target for addressing pancreatitis-related pathophysiology in pancreatic acinar cells.

### 5.3. Prostate Cancer

There exists a limited body of research focused on PAK2′s interactions with metastatic prostate cancer (PCa). One would expect this research pool to be larger, as there are a number of—at least what appears to be—intuitive reasons to study PAK2′s role in prostate cancer development, including the gene’s involvement in actin dynamics, cell survival, and cell proliferation. Additionally, Group I PAKs have been shown to be highly expressed in prostate cancer tissue, while these PAKs are absent in normal prostate tissue [56]. As a note, there do not seem to be any FDA-approved treatments for PCa through therapies targeting PAK2, and this lack of literature should make the gene a promising area of further academic research.

The exact mechanism to activate PAK2 in malignant cancer cells is not well known. That said, it appears that when activated by a proteinase inhibitor in PCa *cells*, PAK2 causes the LIM domain kinase (LIMK) to be phosphorylated. Notably, a study cited that binding of *α*2-macroglobulin to the cell surface-associated (GRP78) receptor in 1-LN prostate cancer activates PAK2, causing the phosphorylation of LIMK. This process apparently depends on receptor tyrosine phosphorylation and the activation of Ras/MAPK and PI 3-kinase [24].

PAK2 has emerged as a significant regulator in prostate cancer (PCa) progression, particularly in the transition from androgen-dependent to androgen-independent stages [57]. A quantitative proteomic profiling of TGF-β-induced epithelial–mesenchymal transition (EMT) in both lymph node carcinoma of the prostate (LNCaP) and PC-3 cell lines identified PAK2 as a key protein involved in apoptosis, cell cycle regulation, and MAPK signaling pathways [58]. Functional studies further demonstrated that PAK2 regulates cell invasion, colony formation, and cell proliferation, positioning it as a potential target for therapeutic intervention in castration-resistant prostate cancer (CRPC). This study underscores the relevance of PAK2 as a clinically actionable biomarker and therapeutic target in PCa [27].

In addition to its involvement in MAPK signaling, PAK2 is regulated by non-coding RNAs such as LINC01006, which modulate its expression through the miR-28-5p/PAK2 axis [59]. A study revealed that PAK2 interacts with Rac1, a key regulator of cell migration, and facilitates lamellipodium extension, a process necessary for directional movement. Moreover, Ras homolog family member H (RhoH), which is normally restricted to hematopoietic cells but is expressed in PCa, regulates Rac1 and PAK2 activity, coupling them to membrane protrusion during migration. High RhoH expression correlates with earlier relapse in PCa patients, further emphasizing the importance of the RhoH–PAK2 axis in PCa progression [60].

Virtanen et al. highlighted the potential of targeting actin regulatory pathways, including PAK2, to inhibit PCa invasion and metastasis, positioning PAK2 as a promising target for therapeutic strategies [61]. Furthermore, a mass spectrometry-based phosphoproteomic study comparing aggressive PCa cell lines, namely PC-3 and PC-3M, revealed that PAK2 was upregulated in highly metastatic cells, with its phosphorylation linked to enhanced tumor migration [62]. These findings collectively emphasize the critical role of PAK2 in PCa progression and its potential as a therapeutic target.

### 5.4. Colorectal Cancer

CRC is a malignant digestive system disease. It is one of the most common cancers in the world. MOB1, a member of a protein family that is highly conserved from yeast to humans, has been found to be involved in the mitotic process of yeast and cell survival, proliferation, differentiation, and organ formation through large tumor suppressor (LATS)/nuclear Dbf2-related (NDR) kinase activity. Studies have also shown MOB1 may be engaged in the regulation of proliferation, apoptosis, migration, and invasion of pancreatic cancer and other malignant tumor cells [26]. Recent research has revealed that MOB1 promotes the malignant progression of colorectal cancer via regulating PAK2. MOB1 and PAK2 are negatively correlated. MOB1 inhibits the malignant progression of colorectal cancer by decreasing PAK2 and thus influences the proliferation and migration of colorectal cancer.

On top of that, it has been suggested that MCM7, a significant subunit of the assumed heteromeric MCM helicase, has a role in the development and spread of tumors and may be a biomarker for a number of human cancers [63]. Zhao et al. further revealed that MCM7 promoted the activation of PAK2, leading to the proliferation and apoptosis of colorectal cancer [35]. Additionally, this work demonstrated that miR-107 regulated MCM7′s impact on apoptosis and proliferation through the PAK2 pathway. The miR-107/MCM7/PAK2 pathway may therefore contribute to the development of cancer.

### 5.5. Ovary Cancer

Cdc42 and Rac1 are important therapeutic targets in ovarian cancer since these two proteins play a critical role in tumor cell migration, adhesion, and invasion [64]. Studies have shown that R-ketorolac inhibits Cdc42 and Rac1 by downregulating PAK2. R-ketorolac was found to dock against Cdc42 and Rac1 GTPase, which reduced stabilization of the bound nucleotides.

On the other hand, a number of CASPs (caspases) members cleave the constitutively active 34 kDa PAK2 C-terminal kinase fragment known as PAK2-p34 [65]. PAK2-p34 recombinant expression caused apoptotic cell death and morphological alteration of apoptosis in a range of cell lines [19,66]. The regulation of cellular death and the execution of programmed cell death are thus mediated by PAK2-p34, and the antiapoptotic activity of PAK2 appears to be converted into a proapoptotic activity of PAK2-p34 by caspase activation triggered by apoptotic stimuli. A study cited that prostasin, a trypsin-like serine peptidase produced in epithelial cells, may be a viable target for the treatment or repression of some ovarian cancers by altering the CASP/PAK2-p34 pathway [67].

Another study revealed that RP11-499E18.1 may have tumor suppressor functions by lowering the nuclear translocation of p-SOX2 and dissociating the relationship between PAK2 and SOX2 in ovarian cancer [68]. Therefore, PAK2–SOX2 interaction and PAK2-p34 recombinant expression are responsible for causing ovarian cancer.

### 5.6. Lung Cancer

Lung cancer is one of the most prevalent cancers in the world. Non-small-cell lung cancer (NSCLC), with two prominent subtypes—lung squamous cell carcinoma (LUSC) and lung adenocarcinoma (LUAD)—is mainly responsible for causing approximately 85–90% of cases of lung cancer [69]. A study conducted by Wang and his co-workers revealed the role of PAK2 in LUSC progression, where they showed that, compared to adjacent normal tissues, higher levels of PAK2 mRNA, DNA copy numbers, and protein were observed in human LUSC tissues [69]. Moreover, they reported that PAK2 improved tumor cell growth, invasion, and migration via regulating actin dynamics through the LIMK1/cofilin signaling pathway. As a consequence, this study indicated that the PAK2/LIMK1/cofilin signaling pathway would be a suitable target for treating LUSC.

Another study showed that after binding to the transcription factor SOX2, which regulates pluripotent stem cells, PAK2 enhances the expression of defective kernel (DEK), which in turn encourages the malignancy of lung squamous cell carcinoma (LSCC) cells and the formation of tumors [70].

### 5.7. Gastric Cancer

One of the most prevalent neoplasms in the digestive tract, gastric cancer is extremely malignant and has a dismal prognosis globally, particularly in Asia and Africa [71]. A study aiming to find a potential correlation between tumor progression and prognosis and PAK2 expression and its phosphorylation state in gastric cancer revealed that the poor prognosis of gastric cancer and accelerated tumor progression may be linked to PAK2 activation [10]. In this study, the expression levels of PAK2 and pSer20PAK2 proteins were both significantly higher (both *p* < 0.001) in gastric cancer tissues compared with normal gastric mucosa. In addition, overexpression of the PAK2 and pSer20PAK2 proteins in patients was significantly linked to negative clinicopathologic factors, such as advanced tumor stage, positive distant metastasis, increased lymph node metastasis, and deeper tumor depth.

On the other hand, a study cited that PAK2 is a cyclin-dependent kinase 12 (CDK12) downstream substrate. CDK12, a gene that drives the growth of gastric cancer in humans, triggers the MAPK signaling pathway by directly binding to and phosphorylating PAK2 at T134/T169, which promotes tumor development and cell division [26]. These findings demonstrate that CDK12/PAK2 may be used as a therapeutic target for gastric cancer patients.

### 5.8. Renal Cancer

Clear-cell renal cell carcinoma (ccRCC) is the most prevalent subtype of kidney cancer, occurring in approximately 75% of cases and known for its high malignancy. It is primarily driven by the loss of the von Hippel–Lindau (VHL) tumor suppressor gene [50]. PAK2 is expected to be substantially activated in ccRCC tumors, according to a comprehensive proteomics investigation conducted by Senturk et al. One study reported that the small GTPases Rac1 and Cdc42 are known to activate PAKs, which are essential for cytoskeletal dynamics, invasion, metastasis, cell death, and proliferation [72]. However, Senturk et al.’s findings indicated that PAK2 especially among all six members of the PAKs is highly functional in ccRCC tumors.

### 5.9. Hepatic Cancer

Long-chain fatty acyl-Coa ligase 4 (ACSL4), a crucial enzyme that transforms fatty acids into fatty acyl-Coa esters, is responsible for causing carcinogenesis [73]. ACSL4 can accelerate the biosynthesis of lipids and encourage the formation of lipid peroxides (LPO), which can cause ferroptosis and promote the development of HCC after chronic liver injury [74]. Wu et al. revealed the precise role of ACSL4 along with PAK2 in causing HCC [75]. In this study, their findings showed that ACSL4 and PAK2 were elevated to varying degrees in HCC tissues and cell lines, and there was a correlation between high expression of these proteins and a poor prognosis for HCC patients. Finally, they reported that ACSL4 expression is highly associated with PAK2 in HCC, and it even transcriptionally upregulates the expression of the PAK2 gene through Sp1, highlighting the function of ACSL4 as a regulator of HCC progression by regulating PAK2. Thus, targeting ACSL4/PAK2 could be useful in drug development and therapy for hepatic cancer.

### 5.10. Miscellaneous

PAK2 plays multifaceted roles throughout hematopoiesis, acting as a negative regulator of granulocyte–monocyte lineage commitment while simultaneously promoting the survival and proliferation of hematopoietic stem cells (HPCs) that remain uncommitted to this lineage [76]. Moreover, PAK2 appears to influence additional hematopoietic processes, including polymorphonuclear neutrophils segmentation and circulation, along with the differentiation and maturation of T and B cells within the thymus and bone marrow.

Deng et al. reported a marked upregulation of PAK2 expression in salivary adenoid cystic carcinoma (AdCC) relative to pleomorphic adenoma (PMA) and normal salivary glands (NSG) [77]. PAK2 was shown to facilitate AdCC cell migration and proliferation, with its proliferative effects potentially associated with cyclin D1 and Ki-67 expression. This study also demonstrated a strong association between PAK2 and p-STAT3 in serial tissue sections and revealed that inhibiting STAT3 leads to a downregulation of PAK2 expression. Although STAT3 directly activates cyclin D1, their findings suggest that STAT3 may also influence cyclin D1 expression through PAK2, introducing an alternative mechanism that could drive AdCC cell proliferation.

In addition, one investigation highlighted the pivotal role of PAK2 in the activation of Wnt/β-catenin signaling in Schwannoma cells, with depletion of PAK2 resulting in diminished levels of active β-catenin, c-Myc, and cyclin D1 [78]. In contrast, in NF2-associated tumors, the abrogation of PAK activity did not influence the Erk or Akt signaling pathways, which are traditionally implicated in PAK-mediated signaling in the context of NF1 [79]. These observations collectively suggest that PAK is a critical regulator of Schwann cell transformation, positioning it as a compelling therapeutic target in both NF1 and NF2. Furthermore, PAK orchestrates multiple signaling cascades in Schwann cells, with distinct pathway variations likely existing between NF1 and NF2, further complicating the cellular landscape of PAK-dependent transformations in these conditions.

**Table 2:** A summary of PAK2′s role and mechanism of action in various types of cancer.

Role	Model	Effects	Target	Mechanism of Action	Reference
*Breast Cancer*					
Tumor Promoter	In vitro, in vivo	Promotes proliferation, migration, and invasion; increases chemoresistance.	STX17	PAK2 enhances autophagy by degrading STX17; inhibition using FRAX486 suppresses autophagy and migration in TNBC cells.	[12]
Prognostic Marker	Clinical	PAK2 overexpression linked to poor prognosis in ER+ breast cancer and TNBC.	PAK2	Drives estrogen-independent growth in ER+ BC and promotes metastasis in TNBC.	[54]
*Pancreatic Cancer*					
Tumor Promoter	In vitro, clinical	Enhances EMT and angiogenesis and reduces differentiation; promotes metastasis and survival.	TGF-β, PKM2, and HSP90	PKM2 phosphorylates PAK2, stabilizing it via HSP90 interaction, preventing ubiquitination and degradation; activates TGF-β signaling to drive EMT.	[49,55]
Tumor Promoter/Pathophysiological	In-vitro	Mediates activation of focal adhesion kinases and MAPKs; modulates PI3K/Akt pathway.	ERK1/2	PAK2 activated ERK1/2, mediating physiological and pathophysiological effects.	[18]
*Prostate Cancer*					
Tumor Promoter	In vitro	Promotes LIMK phosphorylation, actin remodeling, and EMT; linked to metastasis.	LIMK	Activated by α2-macroglobulin binding to GRP78 receptor; depends on Ras/MAPK and PI3K activation.	[24]
Tumor Promoter	In vitro	AR-ligand-treated exosomes promote growth of untreated PCa cells.	Akt1 and PAK2	AR-agonists and antagonists alter exosome protein cargo. Exosomes promote growth and influence the tumor microenvironment (TME) by upregulating pro-proliferative pathways.	[57]
	In vitro (LNCaP, PC-3, and PC-3M)	PAK2 regulates cell invasion, colony formation, and proliferation.	PAK2, Rac1, and RhoH	PAK2 modulates MAPK signaling, EMT, Rac1, and RhoH to drive cell migration, invasion, and proliferation.	[58,60]
	Mass spectrometry (PC-3 and PC-3M)	PAK2 upregulated in metastatic cells, enhancing migration.	PAK2 andAKT1	PAK2 phosphorylation enhances migration in aggressive PCa cell lines, suggesting its role in metastasis.	[61,62]
	Mass spectrometry (xenografts in intact/castrated mice and CRPC cell lines)	PAK2 regulates colony formation, cell invasion, and proliferation in CRPC.	PAK2	PAK2 regulates key processes including cell proliferation, mitotic timing, and invasion in CRPC.	[27]
*Colorectal Cancer*					
Tumor Promoter	In vitro, clinical	Enhances proliferation, migration, and apoptosis resistance; promotes chemoresistance.	MOB1 and MCM7	Negative correlation with MOB1; MCM7 and miR-107 regulate PAK2 to promote malignant progression.	[26,35]
*Ovary Cancer*					
Tumor Promoter	In vitro, clinical	Increases cell migration, invasion, and drug resistance; promotes EMT and survival.	Cdc42, Rac1, and SOX2	PAK2–SOX2 interaction regulates EMT; R-ketorolac suppresses Rac1/Cdc42 pathways to inhibit PAK2 activity.	[65,68]
Pro-apoptotic	CASP-cleavage models	PAK2-p34 promotes apoptotic cell death and regulates programmed cell death via caspase cleavage.	CASPs/PAK2-p34 pathway	Caspase activation cleaves PAK2, converting its anti-apoptotic role to a pro-apoptotic function (PAK2-p34).	[67]
*Lung Cancer*					
Tumor Promoter	In vitro, clinical	Stimulates proliferation, migration, and invasion; enhances tumor growth.	LIMK1	Activates LIMK1/cofilin signaling for cytoskeletal dynamics; PAK2–SOX2 interaction promotes malignancy.	[69,70]
*Gastric Cancer*					
Tumor Promoter	In vitro, clinical	Accelerates tumor progression and metastasis; correlates with poor prognosis.	CDK12	CDK12 phosphorylates PAK2, activating MAPK signaling and promoting tumor growth and cell division.	[10,26]
*Renal Cancer*					
Tumor Promoter	Clinical	Drives invasion, metastasis, and cytoskeletal remodeling.	Rac1 and Cdc42	Rac1 and Cdc42 activate PAK2, facilitating cytoskeletal dynamics and tumor progression.	[72]
*Hepatic Cancer*					
Tumor Promoter	In vitro, clinical	Promotes lipid peroxidation, EMT, and tumor progression; correlates with poor prognosis.	ACSL4 and Sp1	ACSL4 transcriptionally upregulates PAK2 via Sp1; enhances lipid peroxidation, driving ferroptosis and HCC progression.	[75]
*Miscellaneous Cancers*					
Mixed Role	In vitro, clinical	Facilitates hematopoietic stem cell survival; regulates Schwann cell transformation and T/B cell differentiation.	STAT3 and Wnt/β-catenin	PAK2 modulates STAT3 signaling to enhance cyclin D1 expression; activates Wnt/β-catenin signaling in Schwannoma cells.	[77,78]

Cdc42: cell division control protein 42; CRPC: castration-resistant prostate cancer; CASP: caspase; ER+: estrogen receptor-positive breast cancer; ERK1/2: extracellular signal-regulated protein kinases 1 and 2; EMT: epithelial–mesenchymal transition; GRP78: glucose-regulated protein 78; HCC: hepatocellular carcinoma; HSP90: heat-shock protein 90; LIMK: LIM domain kinase; LNCaP: lymph node carcinoma of the prostate; MOB1: Mps one binder 1; MCM7: minichromosome maintenance complex component 7; MAPK: mitogen-activated protein kinase; PCa: prostate cancer; Rac1: Ras-related C3 botulinum toxin substrate 1; SOX2: SRY (sex-determining region Y)-box 2; STX17: Syntaxin 17; TNBC: triple-negative breast cancer cells; STAT3: signal transducer and activator of transcription 3; TGF-β: transforming growth factor-beta.

## 6. Conclusions

While we have only summarized some of the recent research on PAK2′s role in cancer metastasis, the body of research around this gene and its mechanism of action is clearly still at an early stage. More extensive studies are needed to better understand the role of PAK2 in cancer metastasis. Current data suggest that PAK2 may have an important role in promoting metastasis in various cancers. As a protein kinase, PAK2 can be targeted by small molecule inhibitors. Thus, PAK2 is a promising target for new drug discovery to prevent cancer metastasis.

## Figures and Tables

**Table 1:** Summary of PAK2 Overexpression in Cancer Types.

Findings	Implications	References
*Breast cancer*		
PAK2 binds to caspase-7, phosphorylating it at Ser-30, Thr-173, and Ser-239 and reducing its apoptotic activity. Overexpression reverses miR-216a-5p effects, promoting cell proliferation and invasiveness.	Contributes to chemoresistance and poor prognosis by suppressing apoptosis.	[8]
*Hepatocellular carcinoma (HCC)*		
Promotes tumor growth, invasion, and metastasis via TGF-β/Akt signaling and ROS generation.	Potential mediator of early recurrence and metastasis.	[42]
*Head and neck cancer (HNC)*		
Enhances cell proliferation, aerobic glycolysis, and chemoresistance; upregulates c-Myc, which activates PKM2.	Linked to poor clinical outcomes and increased tumor metabolism.	[43]
*Gastric cancer*		
High PAK2 and pSer20PAK2 expression correlates with advanced tumor stage, deeper invasion, and lymph node metastases.	Indicates aggressive disease and poor survival outcomes.	[10]
*Ovarian cancer*		
Elevated p-PAK2 expression drives migration and invasion; associated with activation of the β-catenin/c-Myc/PKM2 pathway and suppression of Nrf2/HO-1 antioxidative response.	Implicated in chemoresistance and worse overall survival.	[44,45,46]
*Oral squamous cell carcinoma (OSCC)*		
Overexpressed in grade III OSCC; correlates with LYN kinase, Slug, and tumor-associated macrophages.	Associated with advanced tumor grade but not overall survival.	[47]
*Colorectal cancer (CRC)*		
Inversely correlated with MOB1 levels; overexpression restores functions disrupted by MCM7 knockdown.	Facilitates proliferation and migration, highlighting interplay with MOB1 and MCM7.	[26,35,48]
*Pancreatic cancer*		
Activates TGF-β signaling to drive pancreatic cancer liver metastasis; correlated with reduced differentiation and enhanced tumor-microenvironment interaction.	Promotes metastatic progression and interaction with the tumor niche.	[49]
*Clear cell renal cell carcinoma (ccRCC)*		
High expression linked to poor survival outcomes.	Serves as a potential prognostic biomarker.	[50]
*Non-small-cell lung cancer (NSCLC)*		
Counteracts tumor-suppressive effects of miR-7-5p, including cell cycle arrest and apoptosis.	Acts as an antagonist of miR-7-5p, driving tumor progression.	[51,52]
*Prostate cancer*		
Regulates SOX2 expression, promoting stemness and treatment resistance.	Enhances tumor plasticity and contributes to castration-resistant progression.	[53]

c-Myc: cellular myelocytomatosis oncogen; LYN: Lck/Yes novel tyrosine kinase; MOB1: Mps one binder 1; MCM7: minichromosome maintenance complex component 7; Nrf2: nuclear factor erythroid 2-related factor 2; PKM2: pyruvate kinase M2; SOX2: SRY (sex-determining region Y)-box 2; TGF-β: transforming growth factor-beta.
